# How to manage food allergy in restaurants, cafeterias and fast food outlets?

**DOI:** 10.1186/2045-7022-1-S1-S20

**Published:** 2011-08-12

**Authors:** Sue Hattersley

**Affiliations:** 1 Food Allergy Branch, Food Standards Agency, London, UK

## 

Food allergen ingredient labelling is now a mandatory requirement in many countries, including those in the European Union, but this only applies to foods sold pre-packaged. There is a general assumption that for foods sold unpackaged, including in catering establishments, it is possible for the consumer to ask the person selling the food about the ingredients used and, based on that information, to make an informed choice about whether or not to buy and consume that food. However it is known that the majority of food allergic reactions occur after eating food that is unpackaged.

Whilst ongoing negotiations in the European Union on a new Food Information Regulation are expected to result in a legal requirement to provide allergen information for foods that are unpackaged, in addition to the existing requirement for pre-packed foods, this is likely to take up to 12 months to complete, and for there to be a transition period before the requirement comes into force. The UK Food Standards Agency recognised the need for best practice guidance on the provision of allergen information for unpackaged foods, and a document was published in 2008 which was aimed at both retailers selling unpackaged foods and caterers. The purpose of this voluntary guidance was to provide practical advice on how such information could be given and to highlight potential problems. This presentation will cover the key messages in the guidance regarding communication between the consumer and the business, the staff training needed and the importance of having access to information about the ingredients used, both in the foods prepared on the premises and also in foods bought in. The presentation will also give some examples of issues that may arise in different types of businesses providing unpackaged foods.

